# Correction: Base recognition by l-nucleotides in heterochiral DNA

**DOI:** 10.1039/c9ra90022e

**Published:** 2019-03-26

**Authors:** Shuji Ogawa, Shun-ichi Wada, Hidehito Urata

**Affiliations:** Osaka University of Pharmaceutical Sciences 4-20-1 Nasahara, Takatsuki Osaka 569-1094 Japan urata@gly.oups.ac.jp +81 (72) 690 1089 +81 (72) 690 1089

## Abstract

Correction for ‘Base recognition by l-nucleotides in heterochiral DNA’ by Shuji Ogawa *et al.*, *RSC Adv.*, 2012, **2**, 2274–2275.

The authors regret that some of the data in the original article were presented incorrectly. Some of the oligonucleotide sequences in the Graphical Abstract, Fig. 2 and Table 1 were originally presented in reverse sequence. The corrected versions of the Graphical Abstract, Fig. 2 and Table 1 are presented below.
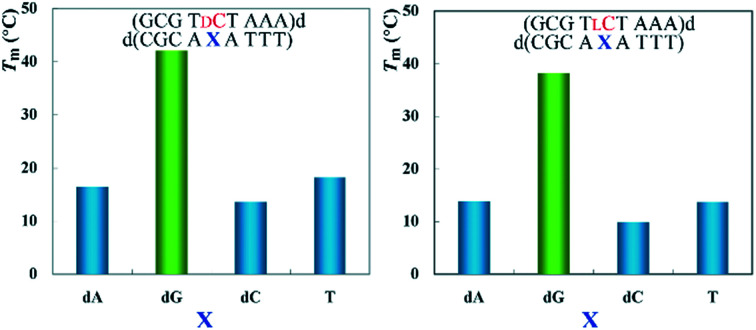


**Fig. 2:**
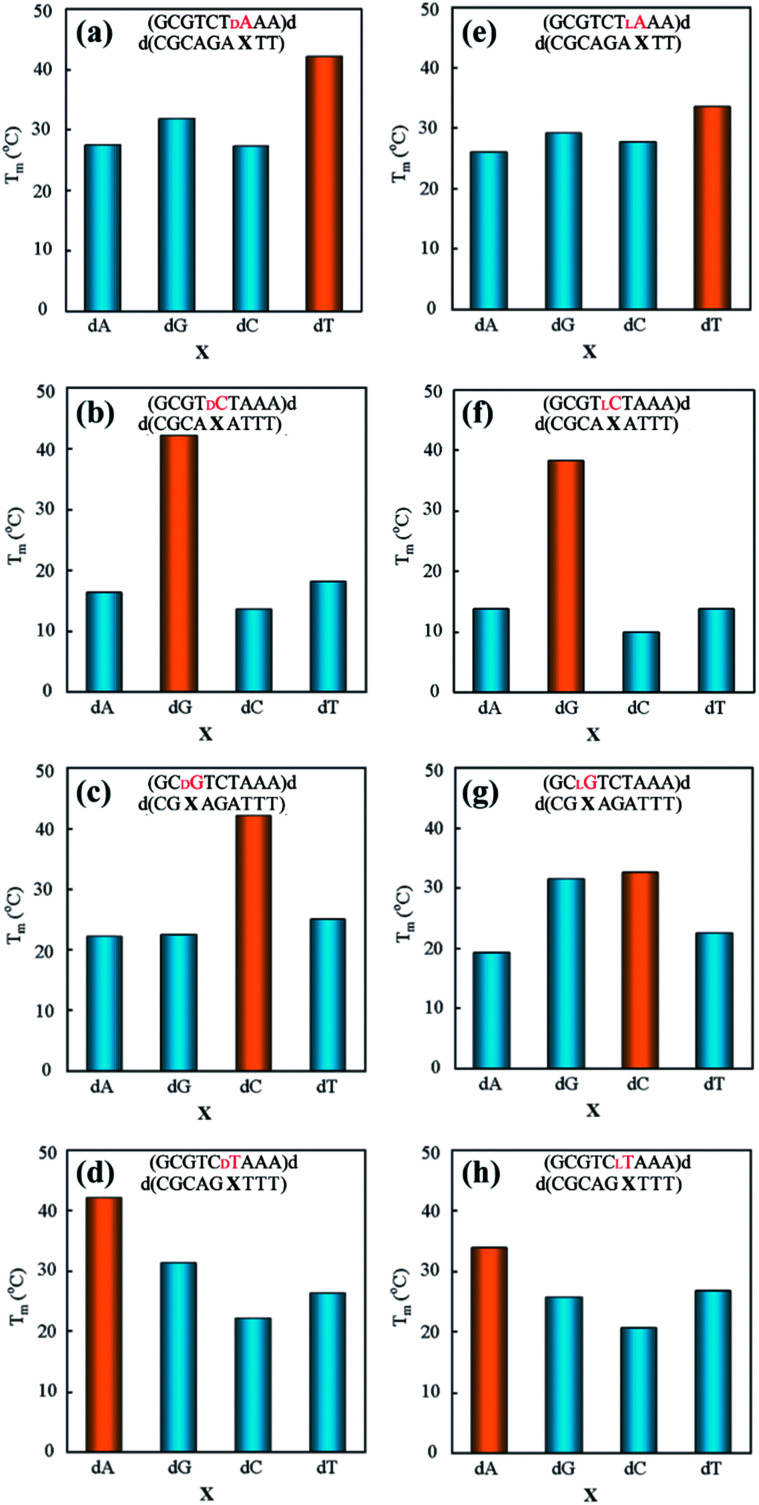
Effects of base pair mismatch of d- (a–d) and l-nucleotide (e–h) on duplex stability. Samples contained 6 mM duplex in 10 mM MgCl_2_, 100 mM NaCl, and 70 mM MOPS (pH 7.1). Yellow bars denote *T*_m_ values of fully matched duplexes, and blue bars denote *T*_m_ values of mismatched duplexes.

**Table 1:** UV-melting points of homo- and heterochiral duplexes^a^

Duplex	Template strand	Complementary strand	*T*_m_ (°C)	Δ*T*_m_^b^ (°C)
**Homochiral strand**				
1	d(AAATCTGCG)	d(CGCAGATTT)	42.1	—

**Heterochiral strand**				
2	d(AA**lA**TCTGCG)	d(CGCAGATTT)	33.6	−8.5
3	d(AAATCT**lG**CG)	d(CGCAGATTT)	32.6	−9.5
4	d(AAAT**lC**TGCG)	d(CGCAGATTT)	38.2	−3.9
5	d(AAA**lT**CTGCG)	d(CGCAGATTT)	33.9	−8.2

a Samples contained 6 μM duplex in 10 mM MgCl_2_, 100 mM NaCl, and 70 mM MOPS (pH 7.1).

b Melting temperature difference from the homochiral duplex.

The Royal Society of Chemistry apologises for these errors and any consequent inconvenience to authors and readers.

## Supplementary Material

